# Analysis of epidemiological characteristics and prognosis based on 1,343 cases of aortic dissection from a regional single center

**DOI:** 10.3389/fcvm.2026.1706284

**Published:** 2026-02-06

**Authors:** Yansong Xu, Cuiqing Huang, Chanyu Huang, Yuewu Wang, Yihuan Luo, Ruiying Wei, Guanbiao Liang

**Affiliations:** 1 Emergency Surgery, First Affiliated Hospital of Guangxi Medical University, Nanning, China; 2 Personnel Section, First Affiliated Hospital of Guangxi Medical University, Nanning, China; 3 Cardiothoracic Surgery, First Affiliated Hospital of Guangxi Medical University, Nanning, China

**Keywords:** aortic dissection, epidemiology, in-hospital mortality, risk factors, Stanford classification

## Abstract

**Objective:**

To analyze the clinical epidemiological characteristics, treatment trends, and risk factors for in-hospital mortality in patients with aortic dissection (AD) at a single center, so as to provide references for early diagnosis and intervention in the emergency department.

**Methods:**

A retrospective analysis was conducted on the medical records of 1343 AD patients admitted between 2011 and 2024. Statistical descriptions were performed for baseline characteristics, clinical manifestations, imaging classification, treatment, and outcomes. Univariate and multivariate logistic regression analyses were employed to identify independent risk factors for in-hospital mortality.

**Results:**

Among the 1,343 patients, 82.7% were male, with a mean age of 52.7 ± 12.4 years; 71.3% had hypertension. Stanford type A and type B AD accounted for 41.7% and 58.3%, respectively. Acute onset was observed in 76.5% of patients, with chest pain (66.4%) being the most common symptom. The onset of AD showed seasonal (peak in winter, especially December) and diurnal (76.6% of cases presented between 12:00 and 23:00) clustering trends. The rate of surgery was 62.0%, showing an increasing trend over the years. The overall mortality rate was 10.0% (95% CI: 8.4%–11.8%), with type A mortality (16.9%, 95% CI: 14.2%–19.9%) significantly higher than that of type B (4.0%, 95% CI: 2.7%–5.7%). Multivariate analysis identified acute onset (OR = 3.484),chest pain (OR = 1.658), increased heart rate (OR = 1.017), Stanford type A (OR = 3.959) and larger false lumen diameter (OR = 1.357) as independent risk factors for in-hospital mortality (all *P* < 0.05). Surgery treatment (OR = 0.194) was a protective factor against in-hospital mortality,

**Conclusion:**

This study indicates that AD predominantly affects middle-aged and elderly males with hypertension, with distinct temporal and seasonal patterns. Acute onset, chest pain, type A dissection, higher heart rate, non-surgical, and larger false lumen diameter significantly increase the risk of in-hospital mortality, highlighting the need for increased vigilance in emergency clinical practice.

## 1 Introduction

Aortic dissection (AD) is a highly lethal cardiovascular emergency, with a globally increasing incidence in recent years. The epidemiological characteristics of AD in the Chinese population differ significantly from those in Western countries, manifesting as an earlier onset age (approximately 10 years younger on average), a higher prevalence but poorer control of hypertension, and more prominent risk factors such as smoking (1–3). Large-scale studies indicate that uncontrolled hypertension can double the risk of AD (4, 5). However, large-sample studies detailing the evolution of clinical characteristics and their association with outcomes in domestic AD patient populations remain relatively scarce. Regarding prognosis, in-hospital mortality for AD remains high (6). Internationally, risk assessment scoring systems like German Registry for Aortic Dissection type A score exist (7), but their applicability in the Chinese population requires further validation. Most current domestic studies have not comprehensively integrated demographic characteristics, clinical manifestations, and imaging data (e.g., Stanford classification, vascular diameters) to analyze their combined impact on prognosis. Therefore, this study analyzed complete clinical data from 1,343 AD patients between 2011 and 2024, aiming to detail its epidemiological characteristics and thoroughly investigate key factors influencing in-hospital outcomes, thereby providing evidence-based support for improving the prognosis of AD patients.

## 2 Materials and methods

### 2.1 Study population

**Figure 1:**
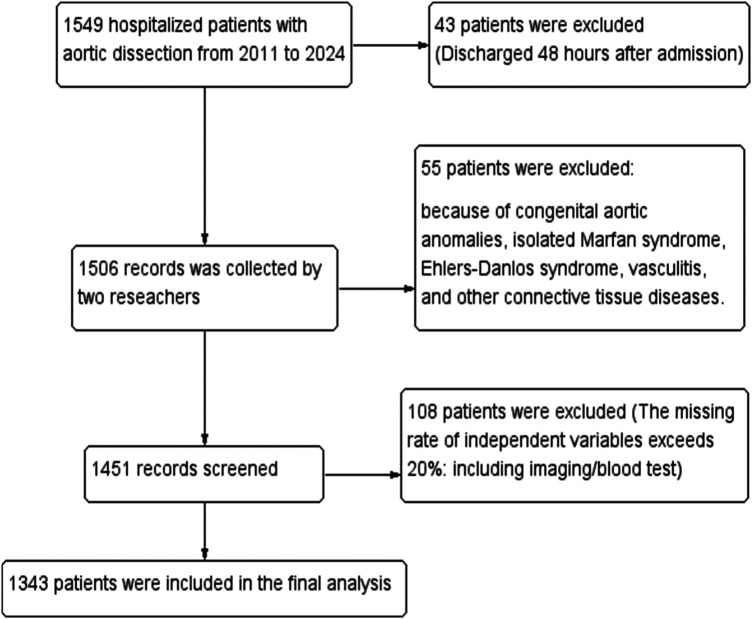
Flowchart of patient with AD enrollment.

The study included patients with imaging-confirmed AD admitted to the First Affiliated Hospital of Guangxi Medical University between January 2011 and December 2024. Medical records were accessed via the Hospital Information System (HIS), and data were extracted independently by two researchers (Figure 1). To mitigate information bias, data abstraction was performed by two independent investigators using a standardized case report form, with discrepancies resolved by a third senior clinician. All imaging parameters were re-measured by two experienced vascular radiologists blinded to the clinical outcomes, and inter-observer agreement was assessed. AD diagnosis was confirmed by computed tomography angiography (CTA), and typing followed the Stanford criteria (8). Medical history included hypertension, diabetes, smoking (≥10 cigarettes/day for ≥5 years), and alcohol consumption [>2 liang (approx. 100 g) of white liquor or >2 bottles of beer per day]. The diagnosis of hypertension is defined according to the 2024 European Hypertension Guidelines (≥140/90 mmHg) (9). The diagnosis of diabetes requires at least two abnormal laboratory values, such as a fasting plasma glucose ≥126 mg/dL (≥7.0 mmol/L), an HbA1c ≥ 6.5% (≥48 mmol/mol), or a random blood glucose ≥200 mg/dL (≥11.1 mmol/L) (10). Death was defined as: (1) All-cause death occurring during the index hospitalization at the study site, regardless of cause. Death must be formally documented in the hospital record. (2) Patient without biological reflex discharged from study hospital with ongoing mechanical circulatory support, mechanical ventilation, or high-dose vasopressors, with a documented plan for immediate transfer to home. According to the guidelines, patients with aortic dissection are classified into acute phase, subacute phase, and chronic phase based on the time of onset (4).

### 2.2 Statistical analysis

Data were processed using Excel and SPSS 26.0. Quantitative data approximately following a normal distribution are expressed as mean ± standard deviation (x¯ ± s), while skewed data are expressed as median (Q1, Q3). Categorical data were compared using the chi-square (*χ*²) test, and continuous data using the *t*-test. Univariate and multivariate logistic regression analyses were used to identify risk factors for mortality. A *P*-value < 0.05 was considered statistically significant.

## 3 Results

### 3.1 Analysis of clinical characteristics of aortic dissection

**Figure 2:**
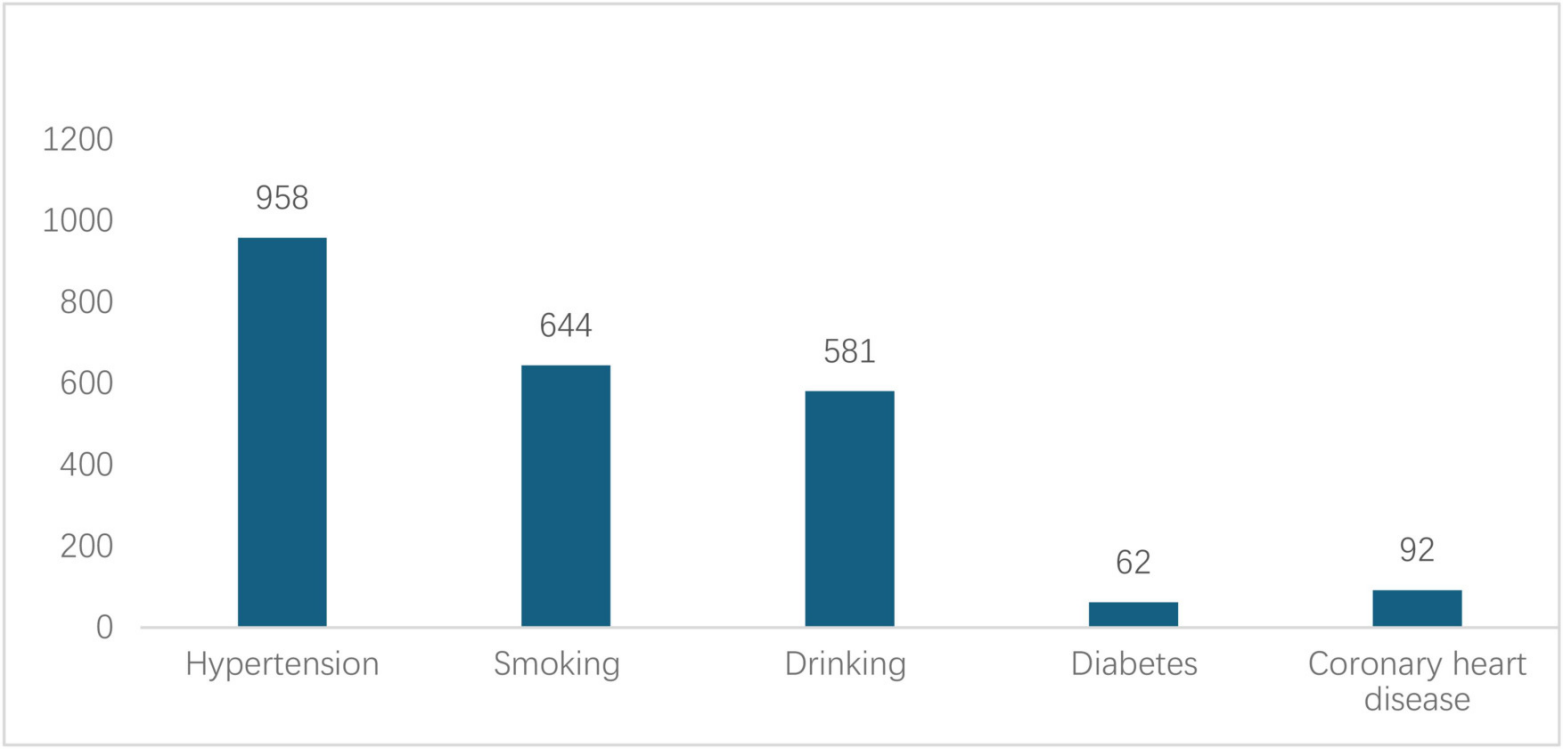
Medical history of patients with AD.

A total of 1343 eligible cases were included, comprising 1110 males and 233 females (male-to-female ratio 4.76:1), with a mean age of 52.7 ± 12.4 years. On admission, 958 patients (71.3%, 95% CI: 68.8%–73.7%) had hypertension; 62 (4.6%) had diabetes mellitus; 644 (48.0%) had a history of smoking; 581 (43.3%) had a history of heavy smoking; and 92 (6.9%) had coronary artery disease (Figure 2). The proportions of Stanford type A and B AD were 41.7% and 58.3%. Age (t = −4.516, P < 0.001), hypertension (*χ*² = 14.828, P < 0.001), and diabetes (*χ*² = 4.289, *P* = 0.038) were significantly associated with Stanford classification. Smoking (*χ*² = 1.907, *P* = 0.167) and alcohol consumption (*χ*² = 3.390, *P* = 0.079) showed no significant statistical association with Stanford classification (Table 1).

**Table 1:** The age, combined hypertension, diabetes, drinking and smoking history of 1343 Stanford type A and B AD patients.

Stanford type	Case	Age	Hypertension	Diabetes	Drinking	Smoking
A	560	51.06 ± 11.03	368	18	258	284
B	783	53.90 ± 11.05	590	44	323	363
t/*χ*²		−4.516	14.828	4.289	3.390	1.907
p-value		0.000	0.000	0.038	0.079	0.167

**Figure 3:**
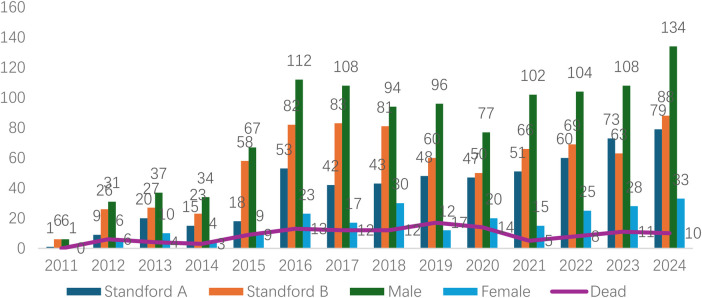
Frequency distribution and death trend of AD (A/B) in male and female patients from 2011 to 2024.

**Figure 4:**
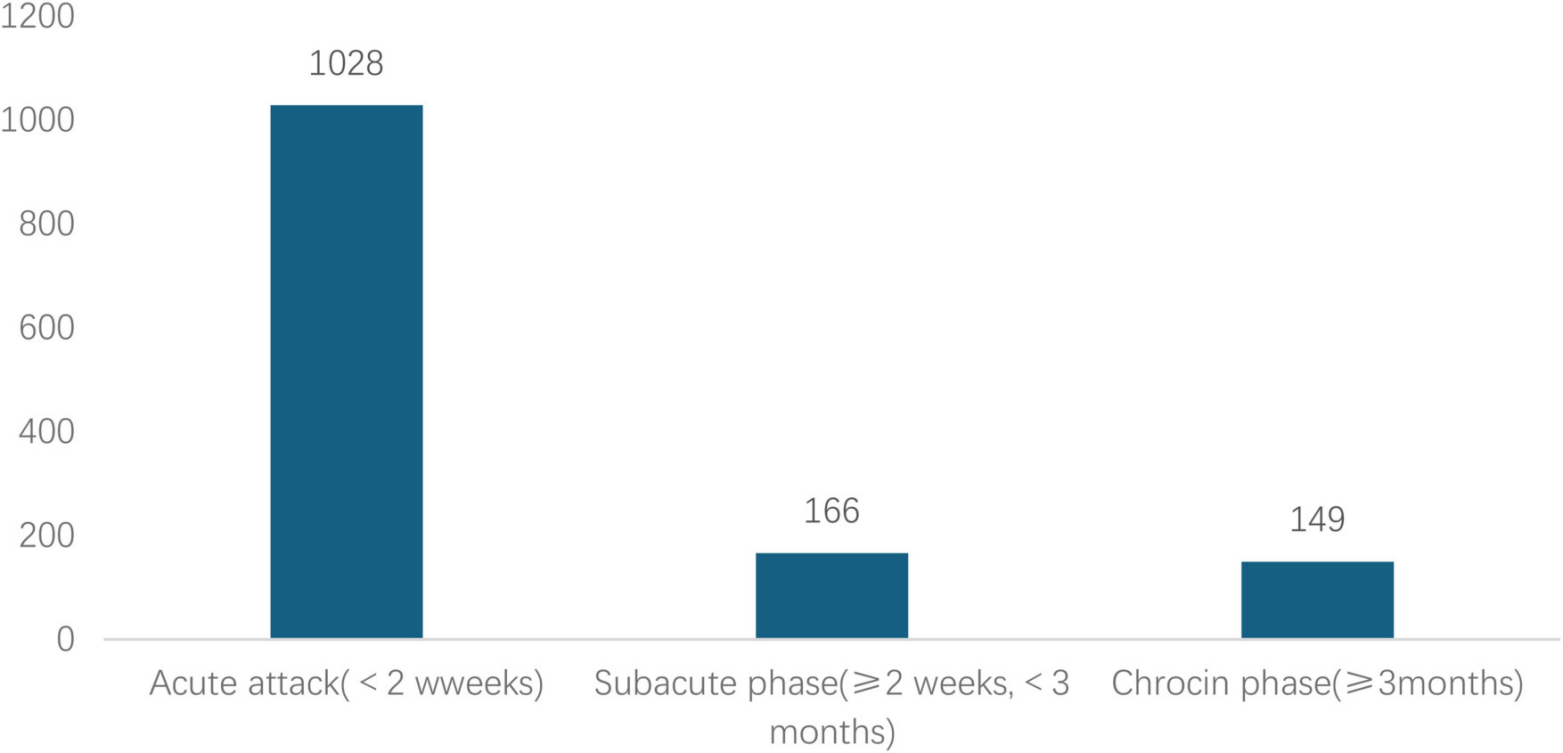
Time from symptom onset to hospital visit for patients with AD.

**Figure 5:**
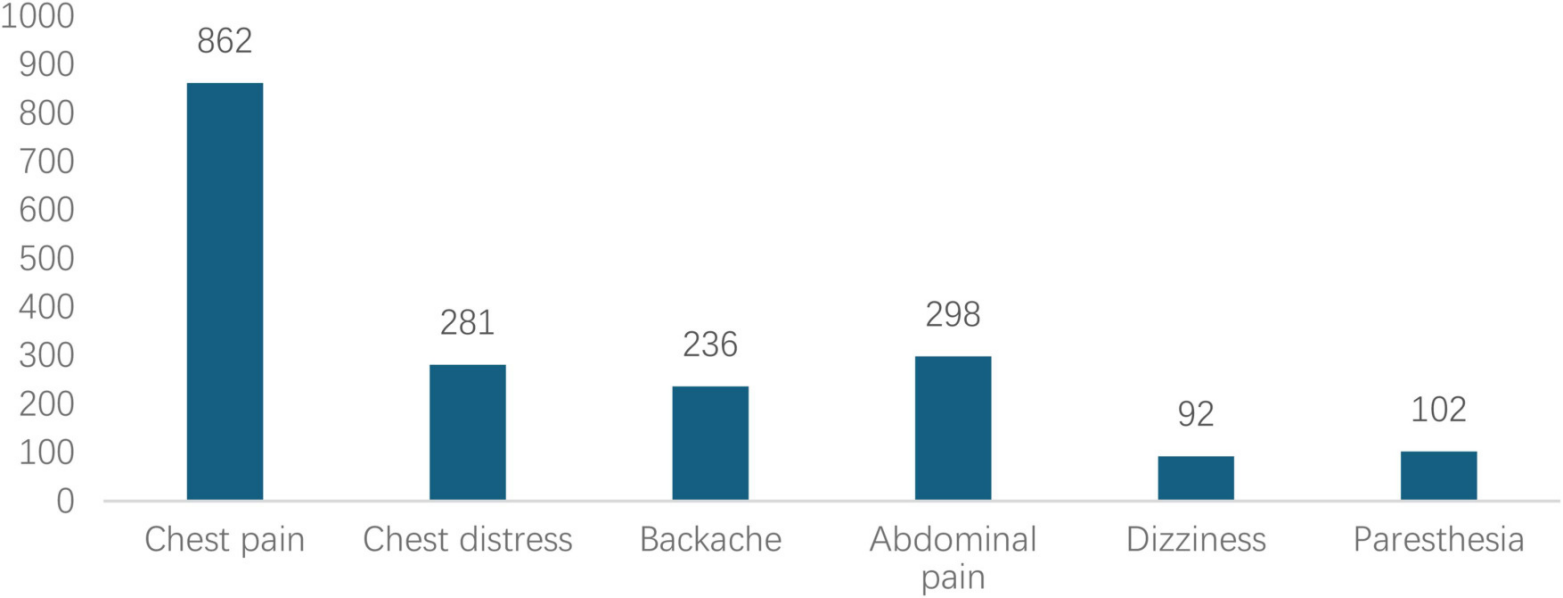
Distribution of chief complaints in patients with AD during their visit.

**Figure 6:**
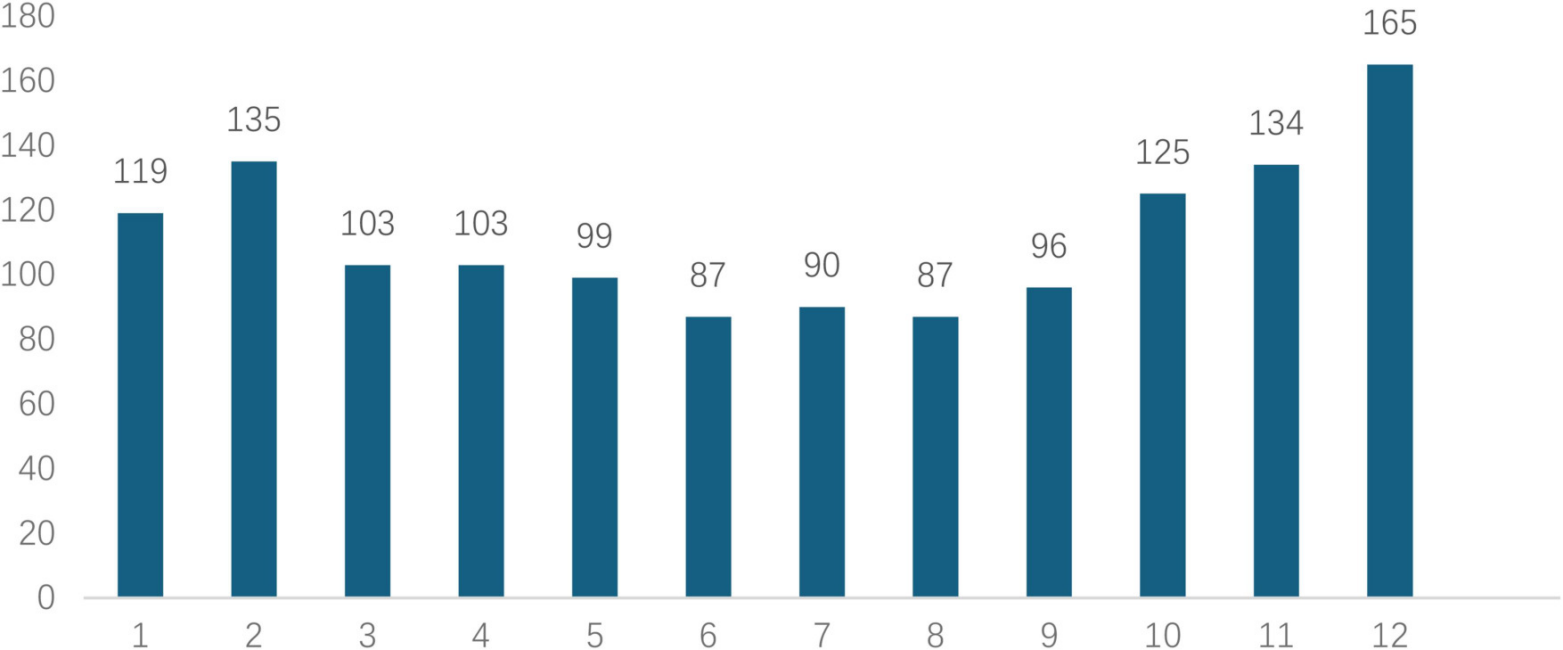
Monthly distribution of visits for patients with AD.

**Figure 7:**
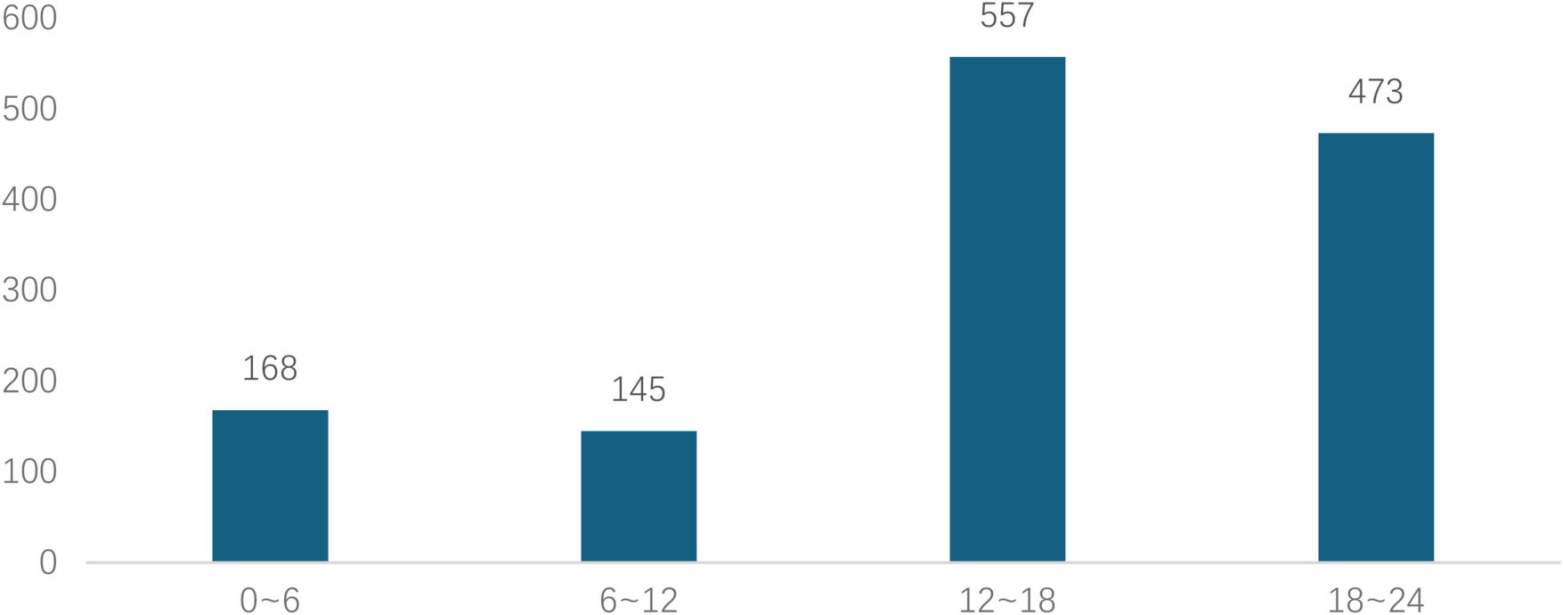
Time to medical consultation after symptom onset (24-hour system) for patients with AD.

**Figure 8:**
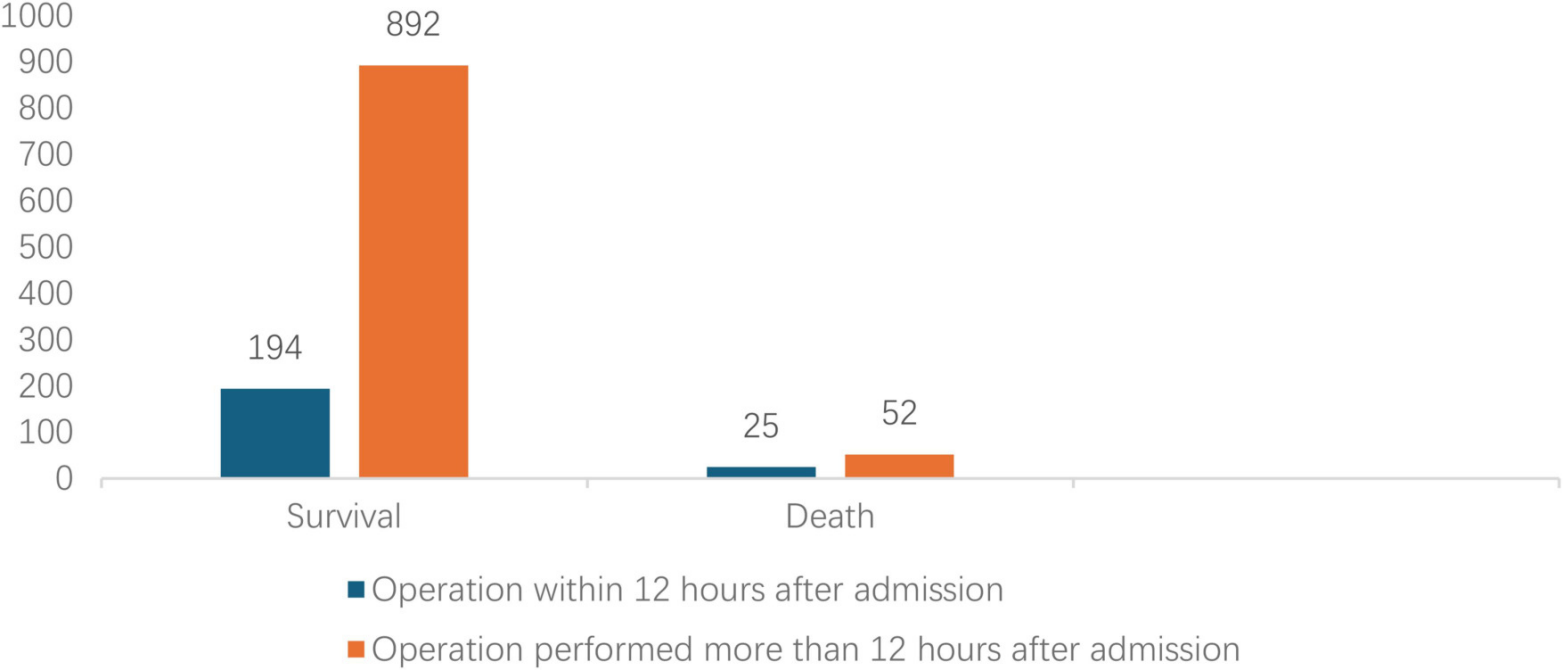
Comparison clinical prognosis of different surgical timing.

Analyzed in 5-year intervals: The overall number of AD cases showed an increasing trend from 2010 to 2024, with growth trends observed from 2011 to 2015 and 2021 to 2024, and a slight decline from 2016-2020. The overall mortality rate was maintained below 10% (Figure 3). Mortality rates were 16.9% for type A and 4.0% for type B dissections. The readmission rate post-AD surgery was 7.4% (87/1163). The majority of AD patients presented with acute onset 76.5% (Figure 4). Regarding presenting symptoms, chest pain was most frequent (892, 66.4%), followed by abdominal pain (Figure 5). AD incidence showed a tendency towards winter and spring seasons, peaking in December (Figure 6). The emergency department admitted the highest number of AD patients between 12:00 and 23:00 daily, accounting for 76.6% of all hospitalized cases (Figure 7). Current AD treatments broadly include conservative medical management, endovascular intervention, and open surgical repair. The number of endovascular interventions has steadily increased, accounting for 62% (701/1130) of the 1130 patients who underwent procedures (Figure 8).

### 3.2 Logistic regression analysis of in-hospital mortality in aortic dissection

Variables included in the analysis were gender, age, chief complaint, past medical history and medication use, imaging features of the dissection, patient vital signs, and treatment method. Univariate logistic regression analysis revealed that acute onset, chest pain, dizziness, conservative management, Stanford type A, faster heart rate, higher systolic blood pressure, higher diastolic blood pressure, larger maximum diameter of the affected vessel, and larger maximum false lumen diameter were significant factors associated with increased in-hospital mortality (*P* < 0.05). Multivariate logistic regression analysis identified acute onset (<2 weeks) (OR = 3.484), chest pain (OR = 1.658), higher heart rate (OR = 1.016), Stanford type A (OR =  3.959) and larger false lumen diameter (OR = 1.357) as independent risk factors for in-hospital mortality (*P* < 0.05). Surgical treatment was a protective factor against in-hospital mortality (OR = 0.194) (Table 2).

**Table 2:** Logistic regression analysis of factors related to aortic dissection mortality from 2011 to 2024.

Variable	Death/total number	Univariate analysis		Multivariate analysis	
		*OR (95%CI)*	*P-value*	*OR (95%CI)*	*P-value*
Gender					
Male	106/1110	1.124 (0.682,1.854)	0.254		
Female	20/233	Ref.			
Age (year)					
∼40	28/186	1.772 (0.218,14.393)	0.592		
40∼,	71/797	0.978 (0.123,7.750)	0.983		
60∼,	26/349	0.805 (0.099,6.535)	0.839		
80∼	1/11	Ref.			
Chest pain					
Yes	99/862	2.182 (1.404,3.391)	0.001	1.658 (1.031,2.666)	*0*.*037*
No	27/481	Ref.		Ref.	
Chest distress					
Yes	25/281	0.929 (0.587,1.470)	0.754		
No	101/1062	Ref.			
Backache					
Yes	17/236	1.407 (0.827,2.394)	0.208		
No	109/1107	Ref.			
Abdominal pain					
Yes	22/298	1.387 (0.859,2.239)	0.181		
No	104/1045	Ref.			
Paresthesia					
Yes	8/102	0.810 (0.384,1.708)	0.580		
No	118/1241	Ref.			
Dizziness					
Yes	3/92	0.309 (0.096,0.991)	0.048	0.310 (0.095,1.017)	0.053
No	123/1251	Ref.		Ref.	
Hypertension					
Yes	88/958	0.924 (0.619,1.378)	0.697		
No	38/385	Ref.			
Diabetes					
Yes	8/62	1.460 (0.679,3.142)	0.333		
No	118/1281	Ref.			
Smoking					
Yes	56/644	0.856 (0.592,1.238)	0.408		
No	70/699	Ref.			
Drinking					
Yes	53/581	0.947 (0.653,1.374)	0.776		
No	73/762	Ref.			
Coronary heart disease					
Yes	8/92	0.914 (0.432,1.935)	0.815		
No	118/1251	Ref.			
Symptom onset time					
<2 weeks	107/1028	4.211 (1.529,11.602)	0.005	3.486 (1.190,10.209)	*0*.*023*
≥2 weeks, <3 months	15/166	3.601 (1.168,11.105)	0.026	3.110 (0.945,10.236)	0.062
≥3months	4/149	Ref.		Ref.	
Heart rate (bpm)	84 ± 16	1.016 (1.006,1.027)	0.002	1.017 (1.006,1.029)	*0*.*003*
SBP (mmHg)	145 ± 25	0.989 (0.982,0.997)	0.005	0.999 (0.988,1.009)	0.798
DBP (mmHg)	83 ± 16	0.980 (0.968,0.991)	0.000	0.993 (0.977,1.010)	0.416
BMI (kg/m2)	23.8 (22.6,26.6)	0.982 (0.940,1.026)	0.422		
Stanford Type					
A	95/560	4.956 (3.251,7.555)	0.000	3.959 (2.484,6.308)	*0*.*000*
B	31/783	Ref.		Ref.	
Maximum diameter of diseased blood vessels (cm)	4.2 ± 1.2	1.157 (1.011,1.325)	0.034	0.913 (0.739,1.127)	0.397
Maximum diameter of false lumen (cm)	2.2 ± 0.9	1.247 (1.064,1.461)	0.006	1.357 (1.064,1.732)	*0*.*014*
Size of interlayer rupture (cm)	1.2 ± 0.5	1.153 (0.850,1.564)	0.359		
50% thrombosis in the false lumen of the sandwich					
Yes	18/286	0.590 (0.352,0.990)	0.046	0.625 (0.356,1.095)	0.101
No	108/1057	Ref.		Ref.	
Aortic vascular calcification					
Yes	75/861	0.806 (0.554,1.173)	0.806		
No	51/282	Ref.			
Aortic dissection with ≥ 2 false lumens					
Yes	19/186	1.116 (0.667,1.868)	0.675		
No	107/1157	Ref.			
Aortic dissection with ≥ 2 ruptures					
Yes	18/177	1.109 (0.655,1.877)	0.700		
No	108/1166	Ref.			
Surgery					
Yes	77/1163	0.190 (0.127,0.282)	0.000	0.194 (0.125,0.301)	*0*.*000*
No	49/180	Ref.		Ref.	

## 4 Discussion

This study analyzed clinical data from 1,343 AD patients over 14 years, systematically elucidating the epidemiological characteristics, clinical features, temporal distribution patterns, evolution of treatment strategies, and independent risk factors for in-hospital mortality. It is representative among similar domestic single-center studies, and its findings have significant implications for understanding the disease burden of AD in the Chinese population, optimizing clinical management pathways, and improving patient outcomes.

A direct comparison of our findings with data from large multinational registries, most notably the International Registry of Acute Aortic Dissection (IRAD), reveals both important consistencies and distinct regional characteristics. Demographically, our cohort was notably younger (mean age 52.7 years) than typical IRAD populations (mean age in the mid-60 s) and had a more pronounced male predominance (M:F ratio 4.76:1 vs. approximately 2:1 in IRAD) (11–13). While hypertension remains the paramount risk factor across all studies, its prevalence and potentially differing control rates may contribute to the earlier disease onset observed in our setting, alongside other population-specific risk factor profiles and potential genetic or environmental influences (2, 13, 14). Regarding management, the significant shift toward thoracic endovascular aortic repair (TEVAR), which accounted for 62% of procedures in our study, aligns with the global trend toward endovascular-first strategies for Stanford type B dissections, as reflected in recent IRAD analyses and guideline recommendations (5, 15). However, the specific rates of surgical versus non-surgical treatment for Stanford type A dissections may vary based on regional expertise and patient selection criteria. In terms of outcomes, the markedly higher in-hospital mortality for Stanford type A (16.9%) compared to Stanford type B (4.0%) dissection is a universal finding, starkly illustrated by IRAD data which reports a mortality of 1%–2% per hour early in Stanford type A dissection (12). Our overall mortality rate of 10.0% and the pattern of risk factors (e.g., Stanford type A classification, larger false lumen diameter) are consistent with the core lessons from international registries (6). The lower mortality for Stanford type B dissection in our cohort may be associated with the high rate of timely TEVAR, underscoring the impact of evolving treatment paradigms on prognosis.

Our results both align with and diverge from patterns reported in major international registries such as IRAD, highlighting population-specific characteristics. For instance, consistent with IRAD, we identified hypertension as the predominant risk factor and Stanford Type A dissection as a major driver of mortality (11, 12). However, our cohort was notably younger (mean age 52.7 vs. mid-60s in IRAD) and had a higher male preponderance (M:F 4.76:1 vs. ∼2:1), underscoring potential differences in risk factor exposure, genetics, or healthcare-seeking behavior in our region (13). The higher incidence in males may be related to hormonal levels (promoting vascular pathological remodeling) and higher exposure rates to factors like smoking and alcohol consumption (14). Hypertension was the most prominent comorbidity in this cohort (71.3%), highlighting uncontrolled hypertension as the most important preventable and controllable risk factor for AD in the Chinese population. Persistent hypertension exerts significant mechanical stress on the aortic wall, leading to medial elastic fiber fragmentation and smooth muscle cell loss, predisposing it to tear under specific triggers (16, 17), Consistent with domestic studies (18), history of hypertension was significantly associated with Stanford classification (p < 0.001). Although the proportion of hypertension was slightly lower in Stanford type A than Stanford type B patients, the absolute number remains substantial, suggesting that strict blood pressure control is the cornerstone of AD primary prevention, regardless of type. Furthermore, smoking history (48%) and heavy alcohol consumption history (43.3%) were significant concomitant risk factors, likely acting synergistically with hypertension by accelerating atherosclerosis and directly damaging vascular endothelium to promote AD occurrence (19). In contrast, the prevalence of diabetes (4.6%) and coronary artery disease (6.9%) was lower, consistent with the pathophysiological mechanism of AD being primarily mechanical damage to the vessel wall rather than atherothrombotic occlusion (20, 21).

Consistent with literature (22, 23), chest pain (66.4%) was the core clinical symptom. Notably, over 30% of patients presented with atypical symptoms such as abdominal pain, back pain, or even lower limb paresthesia, which can easily lead to misdiagnosis, delayed treatment, and missed opportunities for intervention. Multivariate logistic analysis further confirmed that presentation with chest pain was an independent risk factor for in-hospital mortality (OR = 1.658, *p* = 0.037). This might be because chest pain is more frequently associated with the more lethal Stanford type A dissection, and its severity reflects the acuity and seriousness of the tearing process. The proportions of Stanford type A and B dissections were 41.7% and 58.3%, respectively, aligning with some large domestic and international studies (24). Stanford type A patients were younger on average (51.06 vs. 53.90 years, p < 0.001), suggesting that younger individuals with inherent wall defects (e.g., genetic disorders like Marfan syndrome) or more dramatic blood pressure fluctuations might be more susceptible to dissections involving the ascending aorta, which are also more perilous (25).

AD incidence exhibited distinct “dual-peak” distributions: a seasonal peak in winter (culminating in December) and a diurnal peak from noon to night (12:00–23:00, accounting for 76.6%). Temperature drops in winter cause peripheral vasoconstriction, potentially leading to sudden blood pressure surges and increased aortic wall stress (26, 27).The diurnal peak likely correlates with the circadian rhythm of blood pressure (typically higher in the morning and afternoon) and daytime triggers like physical activity and emotional stress (28). These findings suggest that emergency departments should maintain heightened vigilance for AD during these peak periods and seasons, and relevant teams should be prepared for urgent responses. Endovascular intervention (Thoracic Endovascular Aortic Repair; TEVAR) has become the predominant treatment, accounting for 62% (701/1130) of procedures. This aligns with global trends in vascular surgery techniques and is often the preferred treatment for complicated Stanford type B dissections (15). In contrast, this study revealed that the mortality rate of Stanford type B dissection was significantly lower than that of Stanford type A (4% vs. 16.9%).

Multivariate analysis strongly confirmed that surgical treatment served as a protective factor, significantly reducing the risk of death by 80.6% (OR = 0.194, *p* < 0.001), while non-surgical treatment was associated with high risk. Through univariate and multivariate logistic regression analysis, we accurately identified independent risk factors influencing in-hospital mortality in AD patients, which is crucial for clinical risk stratification and prognosis evaluation. Among patients with aortic dissection, the surgical mortality rate during the acute phase was significantly higher than that in the subacute phase. This difference may stem from the pathological features of acute dissection, such as the risk of rapidly progressing tears, susceptibility to organ ischemia or rupture, leading to poorer postoperative outcomes (29). Stanford type A dissections readily involve the pericardium (causing cardiac tamponade), coronary arteries (causing myocardial infarction), aortic valve (causing acute heart failure), and brachiocephalic vessels (causing stroke). Their natural history is extremely perilous, with mortality increasing by 1%–2% per hour within the first 24 hours (12). This result re-emphasizes that for Stanford type A dissection, emergency surgical repair must be organized without delay upon diagnosis; any delay can be fatal. Heart rate is a composite indicator reflecting pain, anxiety, and potential hypovolemia (due to hemorrhage). Sustained tachycardia signifies extreme sympathetic activation, indicating unstable conditions and higher risk of cardiovascular events (30). A large false lumen diameter implies more extensive thrombus formation, more severe true lumen compression and organ mal-perfusion, and also reflects potentially wider tear extent and more severe compromise of aortic wall integrity, increasing the risk of rupture (31).

## 5 Limitations and future directions

This study has several limitations. First, its single-center, retrospective design inherently carries risks of selection bias and information bias. The patient population and treatment patterns may reflect the specific referral patterns, clinical expertise, and institutional protocols of our center, which may limit the generalizability of the findings to other healthcare settings. Second, detailed information such as specific types of antihypertensive medications and pre-admission blood pressure control levels were not extensively collected and analyzed. Finally, this study lacks long-term follow-up data. Therefore, our analysis and conclusions are necessarily confined to in-hospital mortality and complications. We cannot draw inferences regarding long-term survival, re-intervention rates, disease progression, or quality of life beyond the index hospitalization. This limits the comprehensive assessment of the comparative long-term efficacy of different treatment strategies. Future research efforts could focus on: (1) Establishing multicenter, prospective AD registry studies incorporating more detailed variables; (2) Further exploring optimized strategies and perioperative management for type A dissection surgery to further reduce its mortality; (3) Conducting follow-up studies on mid- to long-term complications after TEVAR.

## 6 Conclusion

As a large single-center study from a tertiary cardiovascular hospital in China, the patient population, management protocols, and outcomes may reflect local practices and patient demographics. Therefore, the generalizability of our specific findings, particularly regarding treatment trends and mortality rates, to other regions or healthcare systems should be interpreted with appropriate caution.

## Data Availability

The raw data supporting the conclusions of this article will be made available by the authors, without undue reservation.
